# Transcriptional Control of Honey Bee (*Apis mellifera*) *Major Royal Jelly Proteins* by 20-Hydroxyecdysone

**DOI:** 10.3390/insects9030122

**Published:** 2018-09-19

**Authors:** Paul Winkler, Frank Sieg, Anja Buttstedt

**Affiliations:** 1 Institut für Biologie, Molekulare Ökologie, Martin-Luther-Universität Halle-Wittenberg, Hoher Weg 4, 06120 Halle (Saale), Germany; paul.winkler2@student.uni-halle.de; 2 CuroNZ, 173 Cames Road, Mangawhai 0975, New Zealand; frank@curonz.com; 3 B CUBE—Center for Molecular Bioengineering, Technische Universität Dresden, Arnoldstraße 18, 01307 Dresden, Germany

**Keywords:** juvenile hormone, ecdysone, vitellogenin, social insect, *mrjp*, *apalbumin*, division of labor

## Abstract

One of the first tasks of worker honey bees (*Apis mellifera*) during their lifetime is to feed the larval offspring. In brief, young workers (nurse bees) secrete a special food jelly that contains a large amount of unique major royal jelly proteins (MRJPs). The regulation of *mrjp* gene expression is not well understood, but the large upregulation in well-fed nurse bees suggests a tight repression until, or a massive induction upon, hatching of the adult worker bees. The lipoprotein vitellogenin, the synthesis of which is regulated by the two systemic hormones 20-hydroxyecdysone and juvenile hormone, is thought to be a precursor for the production of MRJPs. Thus, the regulation of *mrjp* expression by the said systemic hormones is likely. This study focusses on the role of 20-hydroxyecdysone by elucidating its effect on *mrjp* gene expression dynamics. Specifically, we tested whether 20-hydroxyecdysone displayed differential effects on various *mrjp*s. We found that the expression of the *mrjp*s (*mrjp1–3*) that were finally secreted in large amounts into the food jelly, in particular, were down regulated by 20-hydroxyecdysone treatment, with *mrjp3* showing the highest repression value.

## 1. Introduction

Molting or ecdysis (from ancient Greek: ἐκδύω (ekduo)) in insects is induced by ecdysteroids, a group of steroid hormones named after the first isolated molting hormone ecdysone [1,2,3]. At the beginning of each molting phase, ecdysteroid levels peak, then decrease before the next phase of ecdysis, and finally ecdysteroid levels drop below a threshold before eclosion of the adult insect (for review see Myers [4]). Corresponding with this, the systemic injection of ecdysteroids causes a concentration dependent delay in eclosion [4,5].

This rule also applies to developing worker honey bees (*Apis mellifera*), where hemolymph ecdysteroid titers decline prior to the pupal molt, increase again in young unpigmented pupae with intense eye pigmentation (~600–800 pg/µL or ~320 pmol/µL of ecdysteroids), and drop to a minimum during cuticle melanization before ecdysis (~50–150 pg/µL and ~60 pmol/µL of ecdysteroids) [6,7,8,9].

A key modulator of ecdysteroid action during honey bee development is juvenile hormone. When juvenile hormone is present, ecdysteroids repeatedly induce larvae to progress through subsequent moltings, but when juvenile hormone is absent, ecdysteroids induce the larval–pupal transition (for review see Hiruma & Kaneko [10]). Furthermore, juvenile hormone and ecdysone regulate several hours before ecdysis the first appearance of the lipoprotein vitellogenin in the hemolymph. The appearance of vitellogenin and ecdysis are delayed by the administration of ecdysone, and juvenile hormone causes them to appear prematurely [11]. Vitellogenin is massively present in the hemolymph of worker honey bees during the first 10 to 15 days of their lives, the time period when these nurse bees feed growing larvae with food jelly [12]. As nurse bees are dependent on protein-rich pollen to eventually produce food jelly, it is not surprising to see that vitellogenin levels in the hemolymph increase greatly after pollen consumption [13]. Strikingly, vitellogenin is a probable direct precursor for the synthesis of food jelly proteins [14,15]. Thus, the production of food jelly proteins depends on the tightly regulated interplay of ecdysteroids, juvenile hormone, vitellogenin, and pollen consumption.

The four most abundant proteins in food jelly belong to the family of major royal jelly proteins (MRJPs), and comprise MRJP1–3 and 5 (for review, see Buttstedt et al. [16]) [17]. These proteins are synthesized by specialized glands located in the head of the honey bee. During the nursing period of a worker bee, gland cells containing acini are known to increase in volume [18], facilitating a proper protein secretion [19]. The functions of MRJPs were largely unknown until recently, but have been gradually elucidated: MRJP1 and 2 have been shown to possess antibacterial activity [20,21]; it is hypothesized that MRJP3 has mainly a nutritional function, due to the occurrence of a repetitive pentapeptide in the sequence delivering high contents of biologically accessible nitrogen to the developing larvae [17,22]; and an oligomeric form of MRJP1 acts as a structural protein, increasing food jelly viscosity via pH-dependent fibril formation in complex with a small RJ protein, apisimin [23]. Overall, the *Apis mellifera* MRJP family consists of nine highly homologue proteins (MRJP1–9) [24,25] that evolved from a single progenitor gene via gene duplication [24]. However, MRJP4 and MRJP6–9 are only secreted in trace amounts into food jelly (for review see Buttstedt et al. [16]) [17]. The tremendous upregulation of *mrjp1–3*, especially, is on the order of between 3000-fold and 15,000-fold, as has been verified in well-fed nurse bees, compared to same aged bees kept on a protein-restricted diet [22]. This circumstance suggests either a tight *mrjp* gene repression until adult hatching and/or a massive induction of gene expression in nurse bees. Although the regulation of *mrjp* expression is not well understood, simultaneous equally weighted transcriptional *mrjp* related network activity involving all of the *mrjp* genes has repeatedly been disproved, because the specific expression profiles among honeybee castes, sexes, developmental stages, and tissues vary markedly [22,24,26]. Because ecdysone and juvenile hormone regulate the emergence of the MRJP precursor vitellogenin in the hemolymph [11], the regulation of *mrjp* expression by the systemic hormones is likely. It has been shown that expression of *mrjp2* is downregulated in nurse bees after administration of either the juvenile hormone analog methoprene, or 20-hydroxyecdysone (20E), the active form of ecdysone [27]. Furthermore, RNAi-mediated knockdown of the ecdysone receptor (EcR) leads to upregulation of *mrjp1* and *9* (3.3- and 2.3-fold, respectively), and downregulation of *mrjp3* (5.5-fold), compared to control bees at eclosion stage [28]. This is a surprising finding, as an opposite regulation of different *mrjp*s under similar conditions has not yet been reported elsewhere. Nevertheless, this indicates that ecdysteroids might play a role in regulating the expression of *mrjp*s. As ecdysteroid concentrations in the hemolymph are high during the late pupal phase (600–800 pg/µL), drop until eclosion (50–150 pg/µL), and are very low in freshly hatched workers (<15 pg/µL) [7,9,29], we expect a repressive role of 20E on the expression of *mrjp*s. If 20E does repress gene expression, freshly hatched worker honey bees that are further supplied with 20E should show a reduced *mrjp* expression compared to control bees. We here investigate whether or not food supplementation with 20E has an inhibitory role on the expression of *mrjp*s. 

## 2. Materials and Methods

### 2.1. Honey Bee Samples

For the collection of newly emerged honey bees (*A. mellifera*), sealed brood frames containing pupae with dark eyes were taken from one colony belonging to the university’s apiary (Halle (Saale), Germany) and placed into an incubator at 34 °C with 60% relative humidity. Immediately after hatching, honey bee workers were assigned to six cages of 60 bees each. The cages were kept at 30 °C and a humidity level of 60%. The bees received water ad libitum and 7 g food dough (49% pollen, 49% icing sugar, 2% water) per day, which either did not contain 20-hydroxyecdysone (20E) (control group—3 cages) or was supplemented with 0.1 mg/g 20E (TCI Deutschland GmbH, Eschborn, Germany) (20E group—3 cages). This concentration was previously shown to be effective. In brief, when employing larval feeding experiments that resulted in pupation and adult emergence, there was only a slight increase in mortality observed (11 and 15% in control and 0.1 mg/g 20E-fed larvae, respectively) [30]. We added pollen to the diet, as previous studies with caged honey bees fed a pollen-free diet showed a strongly reduced expression of *mrjp*s compared to well-fed controls [2]. Consumed food per cage was weighed every day. After three days, 20 bees per cage were frozen in liquid nitrogen and stored at –80 °C until further processing.

### 2.2. RNA Extraction and cDNA Synthesis

Before RNA extraction, the eyes were removed from the bees’ heads, as compound eyes are known to contain PCR inhibitors [31]. Every head was homogenized in 600 µL QIAzol (Qiagen, Hilden, Germany), and RNA extraction was performed according to Chromzynski & Sacchi [32]. Finally, the air-dried RNA pellet was dissolved in 20 µL DEPC water (0.1% (*v*/*v*) diethylpyrocarbonate). RNA quantity and quality were photometrically determined (Nanodrop 1000, Thermo Fisher Scientific, Waltham, MA, USA) and the total RNA was stored at −80 °C. From 1 µg total RNA, cDNA was synthesized according to Buttstedt et al. [22], and purified with the QIAquick PCR purification kit (Qiagen, Hilden, Germany). The cDNA concentration and quality were measured with the Nanodrop 1000, and the cDNA was stored at −20 °C.

### 2.3. Quantitative Real-Time PCR (qPCR)

Quantitative real-time PCR (qPCR) was performed with 1 µL cDNA (adjusted to a concentration of 15 ng/µL DNA) using the SensiMix ^TM^ SYBR^®^ No-Rox Kit (Bioline, Luckenwalde, Germany). Nine bees per cage were analyzed, which were pooled to three cohorts consisting of three bees each. Initially, *ribosomal protein S5α* (*rpS5α*), *actin related protein 1* (*arp1*), *proteasome subunit beta type-1* (*pros26*), and *peptidyl-prolyl cis-trans isomerase-like 2* (*ppil2*) were chosen as reference genes, but only *ppil2* showed a C_t_ (threshold cycle) value standard deviation (SD) below 1 [33] within test conditions, and was therefore used to standardize the expression levels between the samples. The qPCR program was the same for all used primers (Table S1): 10 min denaturation at 95 °C, 40 cycles of 15 s at 95 °C, 30 s annealing at 57 °C, 30 s extension at 72 °C. Subsequently, melting curves (50 °C to 98 °C) were detected, and primer specificity was confirmed with the QIAxcel (Qiagen, Hilden, Germany) to check for specific products and the absence of nonspecific amplicons (Figure S1, Table S1).

### 2.4. Statistics

The C_t_ values of all investigated genes were determined with the CFX Connect TM RealTime System (Bio-Rad, Hemel Hempstead, UK) and the associated software (Bio-Rad CFX Manager v.3.1, Hemel Hempstead, UK). All samples were measured in technical duplicates and subjected to a repeat test cycle when the C_t_ value deviation value exceeded 0.5. The amplification efficiency was determined by serial dilution qPCR (Table S1). Finally, the relative gene expression was determined according to Pfaffl [33], using *ppil2* as the reference gene (Table S2).

To create a heat map, with the inclusion of a dendrogram for genes, relative gene expression values were log transformed and analyzed with the MultiExperiment Viewer (MeV) 4.9.0 [34]. The dendrogram was calculated with Euclidean distance and single-linkage clustering. Presented bootstrap values were calculated in PAST v.3.15 [35] with the same clustering conditions and 1000 bootstrap repetitions.

All statistical analyses were performed with STATISTICA 8.0 (StatSoft, Tulsa, OK, USA). The significance level for all tests was set to α < 0.05. Differences in the control and 20E group regarding consumed food (data normally distributed according to Kolmogorov–Smirnov test, *p* > 0.20) were analyzed with one-way ANOVA with subsequent post-hoc Scheffe test. In addition, partial correlations between consumed food amount and the day after hatching were performed. The raw relative gene expression dataset was not normally distributed (Kolmogorov–Smirnov test, *p* < 0.01). However, normal distribution was obtained by Box-Cox transformation (Kolmogorov–Smirnov test, *p* = 0.086). The influences of variables such as cage identity and 20E administration on gene expression of all nine *mrjp*s were analyzed using a general linear model (GLM), regarding the cage as a random factor, and using a post-hoc Bonferroni test. The expressions of the significantly down-regulated genes (*mrjp1–3*) were correlated to consumed food amount with a Spearman rank correlation. Acini volumes were normally distributed (Kolmogorov–Smirnov test, *p* > 0.05) after log transformation, and thus analyzed by one-way ANOVA with post-hoc Scheffe test.

### 2.5. Acini Volumes

After three days, the acini of 10 bees per treatment were prepared in bee Ringer solution and 20 acini per bee were measured (length and diameter) (Olympus Microscope SZH10 and analySIS software, Olympus, Tokyo, Japan). In addition, ten forager and ten nurse bees were collected from a hive and also measured. Acini volumes were calculated according to Omar et al. [36].

## 3. Results

**Figure 1:**
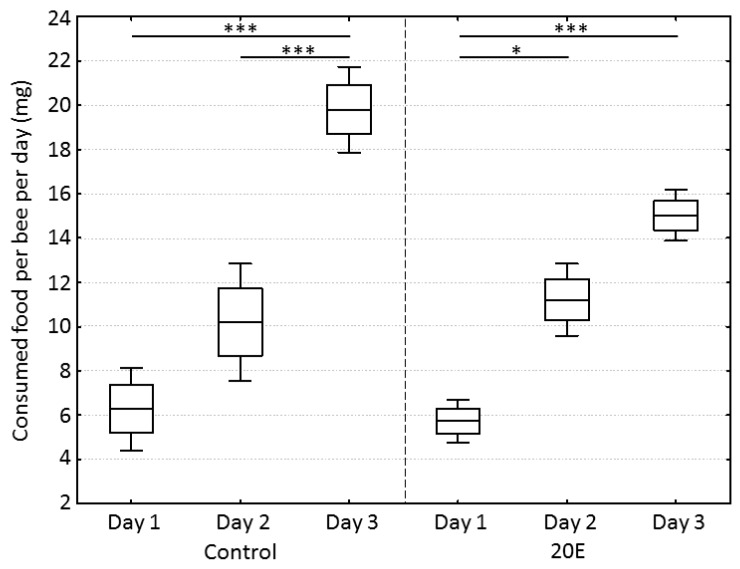
Food consumption per bee per day in mg between control and 20-hydroxyecdysone (20E) group. Boxes show means ± standard errors (SEs) and whiskers show standard deviations (SDs). Statistics were performed using a one-way ANOVA with post-hoc Bonferroni test. Significant differences are indicated by asterisks (*** *p* < 0.001, * *p* < 0.05).

During the incubation time in the cages, bees in the control group and the 20E group consumed an increasing amount of food dough over time (one-way ANOVA, dF = 5, MS = 86.2, F = 27.0, *p* < 0.001) (partial correlations: day-control, r = 0.928, *p* < 0.05; day-20E, r = 0.959, *p* < 0.05), but neither group showed any significant differences regarding consumed food on a specific day (one-way ANOVA, post-hoc Bonferroni test, day 1: *p* = 1.00; day 2: *p* = 1.00, day 3: *p* = 0.10) (Figure 1). In regard to the amount of 20E that was ingested by the bees per day, there was no significant difference between the three replicate cages (mean ± SD; cage 1: 1.06 ± 0.42 µg, cage 2: 0.95 ± 0.48 µg, cage 3: 1.18 ± 0.51 µg) (one-way ANOVA, dF = 2, MS = 0.04, F = 0.19, *p* = 0.83).

**Figure 2:**
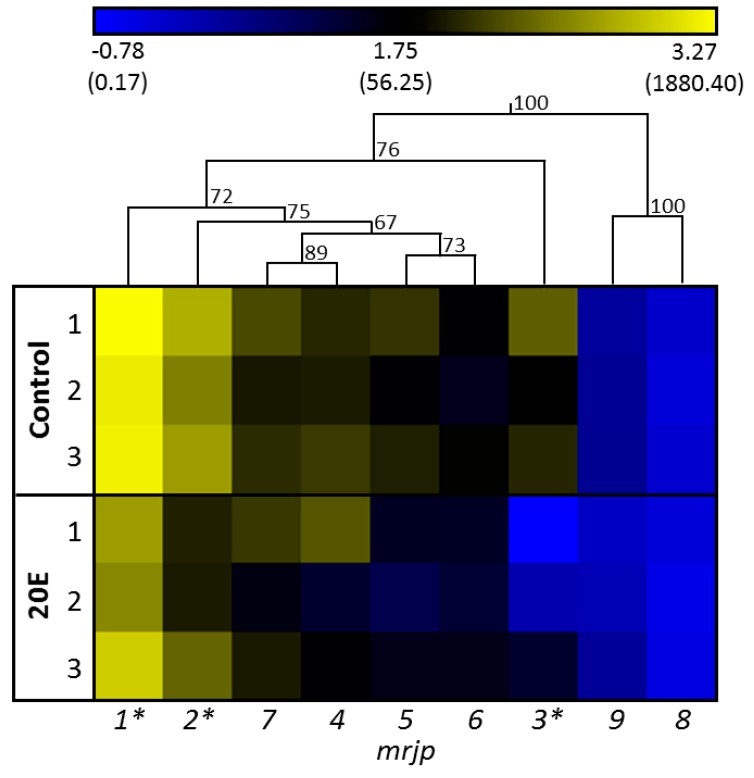
One-way hierarchical clustering analysis heat map and dendrogram of *mrjp* gene expression in the control group compared to the 20E group. Each group comprised three cage replicates, with nine analyzed honey bees per replicate. Gene expression is presented as a color gradient across all samples, from deep blue (lowest) to light yellow (highest). Asterisks indicate significant differences (*p* < 0.05) between control group and 20E group (general linear model (GLM), post-hoc Bonferroni test). Expression values were log transformed for the clustering. Relative gene expression values are shown in brackets.

A significant effect of cage identity in general, or an interaction between gene expression patterns and cages, was not detected (GLM, M = 37.81 and 2.18, F = 4.55 and 1.03, *p* = 0.18 and 0.48, respectively). Figure 2 shows the gene expression of all nine *mrjp*s in relation to the reference gene. In the control group, the expression of *mrjp1* was highest (relative gene expression: 1663 ± 585, mean ± SD), followed by *mrjp2* (491 ± 239), whereas expressions of *mrjp3*, *7*, *4* (121 ± 109 to 108 ± 65, respectively), and *5* and *6* (88 ± 49 and 44 ± 25, respectively), were moderate. Finally, expressions of *mrjp8* and *9* were considerably lower (2.0 ± 0.4 and 0.5 ± 0.2, respectively) than for all other *mrjp*s (Figure 2, Table S2). The effect of 20E was highly specific for the various *mrjp*s. Whereas expressions of *mrjp4–9* did not significantly differ between the control group and the 20E group (post-hoc Bonferroni test, *p* > 0.253), downregulation was moderately high for *mrjp1* and *2* (2.7- and 3.6-fold, respectively; post-hoc Bonferroni test; *mrjp1*, *p* = 0.0225; *mrjp2*, *p* = 0.0231), and substantially higher for *mrjp3* (18.1-fold; post-hoc Bonferroni test, *p* < 0.001) (Figure 2, Table S2). To exclude the possibility that the reduced expressions of *mrjp1–3* were caused by the slightly decreased, albeit not significant, food consumption of the 20E group (15.0 ± 1.2 mg) compared to the control group (19.8 ± 1.9 mg) at day 3 (Figure 1), food consumption was correlated with gene expression (Spearman rank correlation: *mrjp1*, ρ = 0.323, *p* = 0.191; *mrjp2*, ρ = 0.229, *p* = 0.361; *mrjp3*, ρ = 0.348, *p* = 0.157). As the expression of all three genes did not correlate with food consumption, the reduced gene expression in the 20E group can directly be ascribed to the effect of 20E.

The overall effect of 20E on the expression of all *mrjp*s was moderate and not significant (general linear model (GLM), MS = 142.87, F = 17.30, *p* = 0.053). However, there was a significant interaction between genes and treatment (GLM, MS = 10.21, F = 4.81, *p* < 0.01). Indeed, 20E had an effect on some but not all *mrjp*s. This dynamic pattern of 20E effecting *mrjp* gene expression regulation became most prominent for *mrjp3* transcriptional control, as illustrated by the hierarchical clustering, where *mrjp3* formed its own cluster within the group of *mrjp1–7* (Figure 2). Interestingly, the resulting proteins of *mrjp1–3*, showing significant downregulation by 20E, are also the most abundant MRJPs in the food jelly (MRJP1 = 31–35% of total food jelly proteins, MRJP2 = 16–18%, MRJP3 = 24–26%) [17].

**Figure 3:**
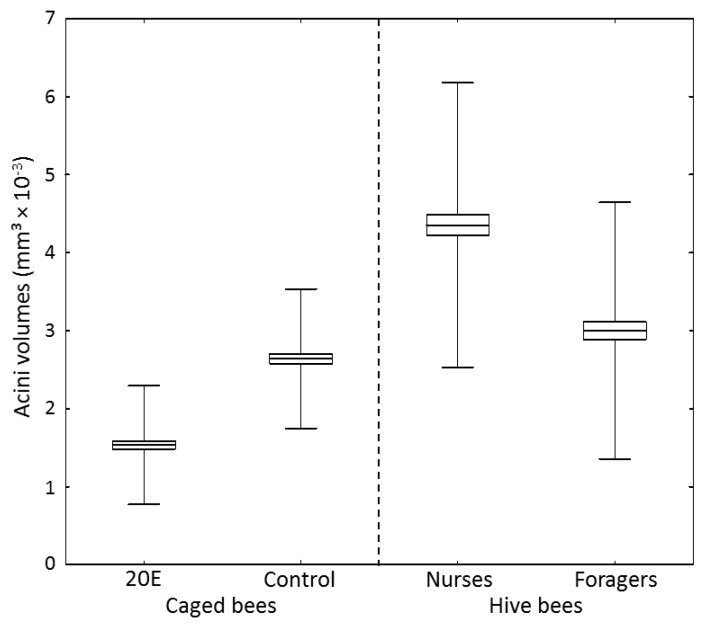
Acini volumes of 10-day-old nurse bees, 24-day-old forager bees, and caged bees of the control group and the 20E group (*n* = 10 bees with 20 acini measured per bee). Acini volumes were calculated as described in [36]. Boxes show means ± SEs and whiskers show SDs.

Food jelly MRJPs are produced within the hypopharyngeal glands, where gland cells containing acini are known to increase in volume during the nursing period of a worker bee [18]. 20E not only reduced food jelly-*mrjp* expression in the caged workers, but had also an effect on acini volumes (Figure 3) (one-way ANOVA, dF = 3, MS = 7.2, F = 187.6, *p* < 0.001). Workers in the 20E group had significantly decreased acini volumes (1.53 ± 0.76 mm^2^ × 10^−3^) compared to workers in the control group (2.64 ± 0.89 mm^3^ × 10^−3^) (post-hoc Scheffe test, *p* < 0.001) (Figure 3). However, the acini volumes of the caged bees in both groups were much smaller than acini volumes of 10-day-old nurse bees sampled from the hive (4.35 ± 1.82 mm^3^ × 10^−3^) (post-hoc Scheffe test, *p* < 0.001). Additionally, though control bees did not significantly differ from forager bees (3.00 ± 1.64 mm^3^ × 10^−3^) (post-hoc Scheffe test, *p* = 0.847), bees fed with 20E had smaller acini volumes than foragers (post-hoc Scheffe test, *p* < 0.001) (Figure 3).

## 4. Discussion

20-Hydroxyecdysone (20E) slightly decreased overall expression of all *mrjp*s and significantly repressed the expression of the most prominent food jelly *mrjp*s (*mrjp1–3*). Whether or not this is a direct hormonal effect cannot be unequivocally verified by analyzing the achieved data set, but three scenarios are possible:

(1) One way in which steroids act in target tissues is by binding to nuclear receptors that recognize thereupon specific DNA sequences, arranged as direct or inverted repeats (for review see Henrich & Brown [37]). Hitherto, ecdysone binds to a heterodimeric receptor subunit of the nuclear ecdysone receptor (EcR) and ultraspiracle (Usp) complex to mediate action [38,39]. Usp binding sites are present in *mrjp* promotor regions [40]. However, the EcR/Usp heterodimer usually activates gene expression in the presence of ecdysone, whereas the absence of ecdysone results in repression (for review see Henrich & Brown [37]). Therefore, a direct repression effect on *mrjp* gene expression, mediated by sole agonist activity at the nuclear ecdysone receptor complex, seems an unlikely explanation for the observed *mrjp* gene expression effects. However, the EcR/Usp dimer might also activate the expression of unknown target genes that in turn repress *mrjp*s.
(2) Besides the classical nuclear receptor action of ecdysone described above, a nongenomic action involving membrane receptors and second messengers (e.g., cyclic AMP) has repeatedly been suggested [41,42]. A first membrane ecdysone receptor was described in 2004 [42], subsequently leading to the identification of primarily G-protein-coupled receptors as being involved in steroid hormone signaling [43]. This membrane receptor-mediated ecdysone signaling can either repress or induce the expression of target genes, and thus might be an option as explanation for *mrjp* regulation.
(3) The production of vitellogenin, being the precursor protein for food jelly proteins [14,15], can be delayed by the administration of ecdysone [11]. Thus, gene expression of the main food jelly *mrjp*s might be reduced simply because of a reduced availability of the precursor” building block” protein.

In a previous study, we showed that *mrjp1–3*, especially, are massively upregulated in brood rearing nurse bees, compared to caged bees of the same age, given a protein-poor diet (between 3000-fold and 15,000-fold) [22]. Caged control group bees in the present study that received pollen in their diet showed *mrjp* expression more similar to nurse bees in the hive, compared to the caged bees without protein in their diet (Table S3). Thus, *mrjp* expression seems to be induced by pollen consumption. Corresponding to these results, the consumption of pollen increases vitellogenin expression and hemolymph vitellogenin levels [13,44]. Although it has been shown that the consumption of pollen also increases hypopharyngeal gland size [45], caged workers of the control group showed rather reduced acini volumes (2.64 ± 0.89 mm^3^ × 10^−3^) compared to nurse bees from the hive (4.35 ± 1.82 mm^3^ × 10^−3^), despite comparable *mrjp* expression. Thus, efficient food jelly protein production is probably dependent on two steps: (1) basic upregulation of *mrjp* expression, in comparison to the reference gene, after pollen consumption, which is most likely dependent on vitellogenin; and (2) increased acini volumes, to enable, in general, the much higher protein production in nurse bee hypopharyngeal glands [46]. This hypopharyngeal gland activation requires direct contact of worker bees with the brood [47], a factor that was not present for the caged bees in this study, which thus possibly explains reduced acini volumes. In addition, the presence of the brood increases, in turn, pollen consumption of nurse bees [48], again increasing vitellogenin levels [13]. This tight interplay between pollen consumption, vitellogenin hemolymph level, and the presence of the brood, leads eventually to a full activation of the food glands.

As the concentrations of ecdysteroids are high during the pupal phase of the bees [6,7,9], it might be that the hormone represses (directly or indirectly) the production of *mrjp*s during this phase. Subsequently, ecdysteroids decrease in the hemolymph, probably leading to cessation of *mrjp* gene repression, accompanied by an induction of expression due to pollen consumption after hatching. This circumstance is most important for MRJP1–3, as these proteins are the main proteins in RJ [17], and the temporal transcriptional activation within the nurse bees is required to provide food for the larvae.

## 5. Conclusions

We here show that food supplementation with 20E has indeed an inhibitory role on the expression of the most abundant food jelly *mrjps* (*mrjp1-3*), with *mrjp3* showing the highest downregulation (18-fold), while having no effect on *mrjp4-9*.

## Supplementary Materials

The following are available online at http://www.mdpi.com/2075-4450/9/3/122/s1. Figure S1: Analysis of qPCR product specificity, Table S1: Primer and product characteristics for qPCR, Table S2: Relative gene expression of all *mrjp*s, Table S3: Comparison between C_t_ values.

## References

[1] Butenandt A., Karlson P. (1954). Über die Isolierung eines Metamorphose-Hormons der Insekten in kristallisierter Form. Z. Naturforsch..

[2] Karlson P. (1956). Chemische Untersuchungen über die Metamorphosehormone der Insekten. Ann. Sci. Nat. Zool. Biol. Anim..

[3] Karlson P., Hoffmeister H., Hoppe W., Huber R. (1963). Zur Chemie des Ecdysons. Justus Liebigs Ann. Chem..

[4] Myers E.M. (2003). The circadian control of eclosion. Chronobiol. Int..

[5] Schwartz L.M., Truman J.W. (1983). Hormonal control of rates of metamorphic development in the tobacco hornworm *Manduca sexta*. Dev. Biol..

[6] Amdam G.V., Page R.E., Fondrk M.K., Brent C.S. (2010). Hormone response to bidirectional selection on social behavior. Evol. Dev..

[7] Pinto L.Z., Hartfelder K., Gentile Bitondi M.M., Simões Z.L.P. (2002). Ecdysteroid titers in pupae of highly social bees relate to distinct modes of caste development. J. Insect Physiol..

[8] Rachinsky A., Strambi C., Strambi A., Hartfelder K. (1990). Caste and metamorphosis: Hemolymph titers of juvenile hormone and ecdysteroids in last instar honeybee larvae. Gen. Comp. Endocrinol..

[9] Zufelato M.S., Bitondi M.M.G., Simões Z.L.P., Hartfelder K. (2000). The juvenile hormone analog pyriproxyfen affects ecdysteroid-dependent cuticle melanization and shifts the pupal ecdysteroid peak in the honey bee (*Apis mellifera*). Arthropod Struct. Dev..

[10] Hiruma K., Kaneko Y. (2013). Hormonal regulation of insect metamorphosis with special reference to juvenile hormone biosynthesis. Curr. Top. Dev. Biol..

[11] Barchuk A.R., Bitondi M.M.G., Simões Z.L.P. (2002). Effects of juvenile hormone and ecdysone on the timing of vitellogenin appearance in hemolymph of queen and worker pupae of *Apis mellifera*. J. Insect Sci..

[12] Engels W. (1974). Occurrence and significance of vitellogenins in female castes of social hymenoptera. Am. Zool..

[13] Bitondi M.M.G., Simoes Z.L.P. (1996). The relationship between level of pollen in the diet, vitellogenin and juvenile hormone titres in Africanized *Apis mellifera* workers. J. Apic. Res..

[14] Amdam G.V., Norberg K., Hagen A., Omholt S.W. (2003). Social exploitation of vitellogenin. Proc. Natl. Acad. Sci. USA.

[15] Seehuus S.-C., Norberg K., Krekling T., Fondrk K., Amdam G.V. (2007). Immunogold localization of vitellogenin in the ovaries, hypopharyngeal glands and head fat bodies of honeybee workers, *Apis mellifera*. J. Insect Sci..

[16] Buttstedt A., Moritz R.F.A., Erler S. (2014). Origin and function of the major royal jelly proteins of the honeybee (*Apis mellifera*) as members of the yellow gene family. Biol. Rev..

[17] Schmitzová J., Klaudiny J., Albert S., Schröder W., Schreckengost W., Hanes J., Júdová J., Simúth J. (1998). A family of major royal jelly proteins of the honeybee *Apis mellifera* L.. Cell. Mol. Life Sci..

[18] Kratky E. (1931). Morphologie und Physiologie der Drüsen in Kopf und Thorax der Honigbiene (*Apis mellifica* L.). Z. Wiss. Zool..

[19] Patel N.G., Haydak M.H., Gochnauer T.A. (1960). Electrophoretic components of the proteins in honeybee larval food. Nature.

[20] Bíliková K., Mirgorodskaya E., Bukovská G., Gobom J., Lehrach H., Šimúth J. (2009). Towards functional proteomics of minority component of honeybee royal jelly: The effect of post-translational modifications on the antimicrobial activity of apalbumin2. Proteomics.

[21] Vezeteu T.V., Bobiş O., Moritz R.F.A., Buttstedt A. (2017). Food to some, poison to others-honeybee royal jelly and its growth inhibiting effect on European Foulbrood bacteria. MicrobiologyOpen.

[22] Buttstedt A., Moritz R.F.A., Erler S. (2013). More than royal food-Major royal jelly protein genes in sexuals and workers of the honeybee *Apis mellifera*. Front. Zool..

[23] Buttstedt A., Mureşan C.I., Lilie H., Hause G., Ihling C.H., Schulze S.H., Pietzsch M., Moritz R.F.A. (2018). How honeybees defy gravity with royal jelly to raise queens. Curr. Biol..

[24] Drapeau M.D., Albert S., Kucharski R., Prusko C., Maleszka R. (2006). Evolution of the yellow/major royal jelly protein family and the emergence of social behavior in honey bees. Genome Res..

[25] Helbing S., Lattorff H.M.G., Moritz R.F.A., Buttstedt A. (2017). Comparative analyses of the major royal jelly protein gene cluster in three Apis species with long amplicon sequencing. DNA Res..

[26] Chan Q.W., Chan M.Y., Logan M., Fang Y., Higo H., Foster L.J. (2013). Honey bee protein atlas at organ level resolution. Genome Res..

[27] Ueno T., Nakaoka T., Takeuchi H., Kubo T. (2015). Changes in the Gene Expression Profiles of the Hypopharyngeal Gland of Worker Honeybees in Association with Worker Behavior and Hormonal Factors. PLoS ONE.

[28] Mello T.R.P., Aleixo A.C., Pinheiro D.G., Nunes F.M.F., Bitondi M.M.G., Hartfelder K., Barchuk A.R., Simões Z.L.P. (2014). Developmental regulation of ecdysone receptor (EcR) and EcR-controlled gene expression during pharate-adult development of honey bees (*Apis mellifera*). Front. Genet..

[29] Hartfelder K., Bitondi M.M.G., Santana W.C., Simões Z.L.P. (2002). Ecdysteroid titer and reproduction in queens and workers of the honey bee and of a stingless bee: Loss of ecdysteroid function at increasing levels of sociality?. Insect Biochem. Mol. Biol..

[30] Rharrabe K., Bouayad N., Sayah F. (2009). Effects of ingested 20-hydroxyecdysone on development and midgut epithelial cells of *Plodia interpunctella* (Lepidoptera, Pyralidae). Pestic. Biochem. Physiol..

[31] Boncristiani H., Li J., Evans J.D., Pettis J., Chen Y. (2011). Scientific note on PCR inhibitors in the compound eyes of honey bees, *Apis mellifera*. Apidologie.

[32] Chromzynski P., Sacchi N. (1987). Single-step method of RNA isolation by acid guanidinium thiocyanate-phenol-chloroform extraction. Anal. Biochem..

[33] Pfaffl M.W. (2001). A new mathematical model for relative quantification in real-time RT-PCR. Nucleic Acids Res..

[34] Howe E., Holton K., Nair S., Schlauch D., Sinha R., Quackenbush J., Ochs M.F., Casagrande J.T., Davuluri R.V. (2010). Mev: Multiexperiment viewer. Biomedical Informatics for Cancer Research.

[35] Hammer O., Harper D.A.T., Ryan P.D. (2001). PAST: Paleontological statistics software package for education and data analysis. Palaeontol. Electron..

[36] Omar E., Abd-Ella A.A., Khodairy M.M., Moosbeckhofer R., Crailsheim K., Brodschneider R. (2017). Influence of different pollen diets on the development of hypopharyngeal glands and size of acid gland sacs in caged honey bees (*Apis mellifera*). Apidologie.

[37] Henrich V.C., Brown N.E. (1995). Insect nuclear receptors: A developmental and coparative perspective. Insect Biochem. Mol. Biol..

[38] Barchuk A.R., Maleszka R., Simões Z.L.P. (2004). *Apis mellifera* ultraspiracle: CDNA sequence and rapid upregulation by juvenile hormone. Insect Mol. Biol..

[39] Yao T.P., Segraves W.A., Oro A.E., McKeown M., Evans R.M. (1992). Drosophila ultraspiracle modulates ecdysone receptor function via heterodimer formation. Cell.

[40] Malecová B., Ramser J., O’Brien J.K., Janitz M., Júdová J., Lehrach H., Simúth J. (2003). Honeybee (*Apis mellifera* L.) mrjp gene family: Computational analysis of putative promoters and genomic structure of mrjp1, the gene coding for the most abundant protein of larval food. Gene.

[41] Applebaum S.W., Gilbert L.I. (1972). Stimulation of adenyl cyclase in pupal wing epidermis by β-ecdysone. Dev. Biol..

[42] Elmogy M., Iwami M., Sakurai S. (2004). Presence of membrane ecdysone receptor in the anterior silk gland of the silkworm *Bombyx mori*. Eur. J. Biochem..

[43] Wang D., Zhao W.-L., Cai M.-J., Wang J.-X., Zhao X.-F. (2015). G-protein-coupled receptor controls steroid hormone signaling in cell membrane. Sci. Rep..

[44] Di Pasquale G., Salignon M., Le Conte Y., Belzunces L.P., Decourtye A., Kretzschmar A., Suchail S., Brunet J.-L., Alaux C. (2013). Influence of pollen nutrition on honey bee health: Do pollen quality and diversity matter?. PLoS ONE.

[45] Hrassnigg N., Crailsheim K. (1998). Adaptation of hypopharyngeal gland development to the brood status of honeybee (*Apis mellifera* L.) colonies. J. Insect Physiol..

[46] Knecht D., Kaatz H.H. (1990). Patterns of larval food production by hypopharyngeal glands in adult worker honey bees. Apidologie.

[47] Huang Z.-Y., Otis G.W., Teal P.E.A. (1989). Nature of brood signal activating the protein synthesis of hypopharyngeal gland in honey bees, *Apis mellifera* (Apidae: Hymenoptera). Apidologie.

[48] Hrassnigg N., Crailsheim K. (1998). The influence of brood on the pollen consumption of worker bees (*Apis mellifera* L.). J. Insect Physiol..

