# Supplementary material for: Transcriptional Control of Honey Bee (Apis mellifera) Major Royal Jelly Proteins by 20-Hydroxyecdysone
Source: Insects. 2018 Sep 19;9(3):122. doi: 10.3390/insects9030122 (PMC6163268; doi:10.3390/insects9030122)
Supplement: Supplementary file 1 [file insects-09-00122-s001.pdf]

Supporting information: Transcriptional control of honey bee (*Apis mellifera*) *major royal jelly proteins* by 20-hydroxyecdysone.

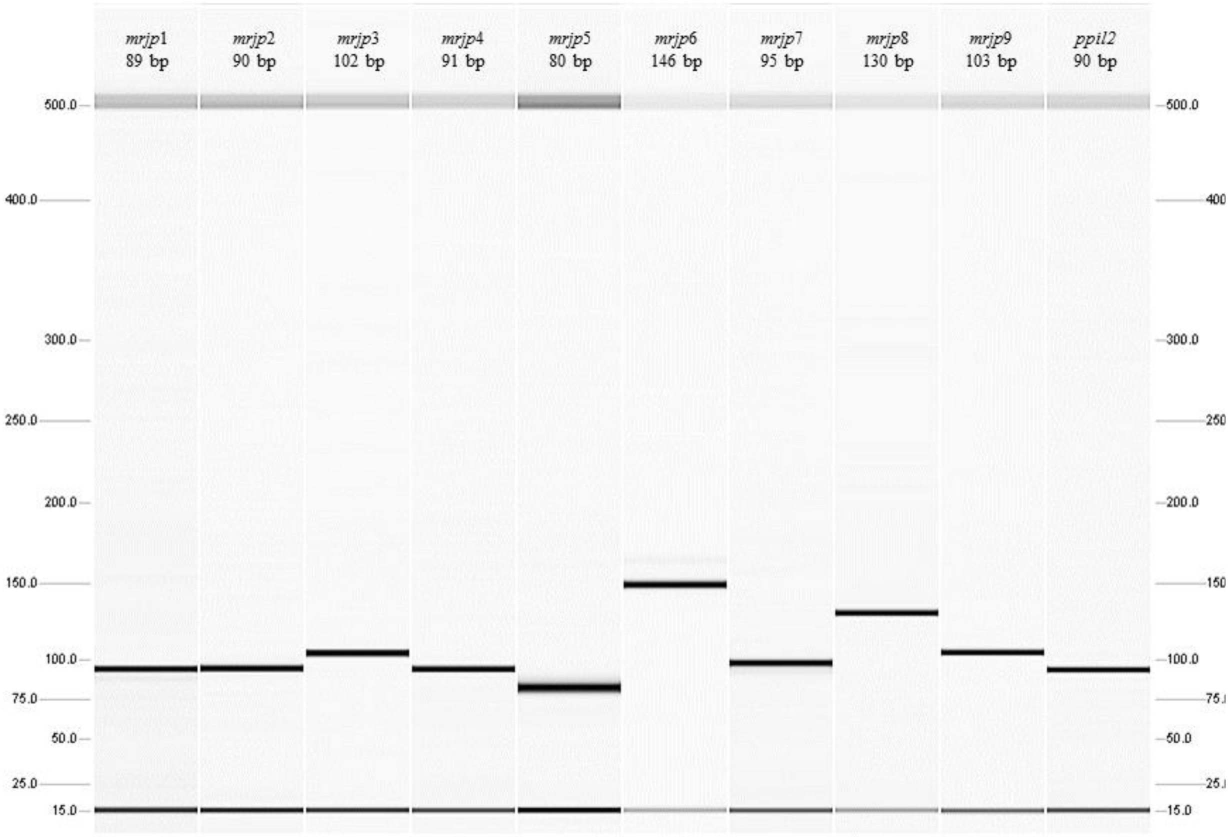

Figure S1: Analysis of qPCR product specificity.

Table S1: Primer and product characteristics for qPCR.

Name		Primer Sequence (5'-3')	Size (bp)	Product T_m (°C)	PCR efficiency	GenBank Gene ID	Reference
mrjp1	F	TGACATACATTACGAAGGAGTCCA	89	76.0	1.95	406090	
	R	ATCCGAAGAAGAGAACGCCA					
mrjp2	F	CGTCCAATACCAAGGATCCGAA	90	77.5	1.92	406091	
	R	ACAAGTCCGACGAAGAGGAC					
mrjp3	F	TGGACAGATGGCGTGATAAGAC	102	76.5	1.96	406121	
	R	GAGGTCCACCTTTGCCCTTT					
mrjp4	F	AGACAAAATATCGATGTCGTAGCTC	91	76.9	1.98	406133	
	R	TGCCAGATTGTGGAACGTTTT					
mrjp5	F	AGAAAATATGGATATGGTCGCTCAG	80	76.5	1.98	406116	Buttstedt et al., 2013
	R	ATTGTGGGAGATGCATCATTTTCA					
mrjp6	F	ACAAAATATGGATATGGTCGCTCAG	146	75.3	1.91	406149	
	R	TCTGCATTCTGTTACTTAAAGCCAA					
mrjp7	F	AGAAAATACCGATATGGTCGCC	95	76.0	1.98	409555	
	R	CAATAATGACGATATGTGGGAGCA					
mrjp8	F	TGGACTCAAGCATCGGCTAA	130	78.5	1.92	406067	
	R	TGGCAACCACTTCGATATTTCTT					
mrjp9	F	TCGGAAACTGGTGCTCTCTT	103	80.0	1.93	409873	
	R	TGGCGACTATTTCAATATTCCTTCT					
ppil2	F	TTTAATGCGGCACACTATTCTACT	90	77.7	1.91	550966	this study
	R	AACTGCTGCTTGATGTGTAGTTTC					

Table S2: Relative gene expression of all *mrjps* across both caged groups (control (C) and 20E (E)).

	mrjp1	*mrjp2*	*mrjp3*	*mrjp4*	*mrjp5*	*mrjp6*	*mrjp7*	*mrjp8*	*mrjp9*
C1-1	892.14	355.41	78.08	91.21	74.26	34.59	116.39	0.52	1.68
C1-2	2033.02	695.39	171.84	189.70	116.12	33.79	218.00	0.49	1.24
C1-3	2716.05	891.68	376.96	25.11	173.26	74.61	141.58	0.81	2.05
C2-1	1086.47	434.07	77.78	118.68	76.32	35.13	104.34	0.46	1.87
C2-2	1641.88	36.62	3.82	7.13	6.10	13.58	20.53	0.36	2.14
C2-3	1743.97	520.83	78.60	140.86	64.27	28.87	130.93	0.51	2.44
C3-1	1758.15	504.27	157.61	121.70	117.69	79.43	108.62	0.53	2.34
C3-2	1039.27	375.17	94.12	77.65	46.60	26.07	82.22	0.39	1.60
C3-3	2058.76	602.29	51.31	195.16	115.01	73.15	139.50	0.76	2.56
E1-1	647.89	131.39	0.27	56.31	20.87	31.49	176.00	0.65	0.82
E1-2	324.30	69.96	0.10	19.94	15.48	4.78	82.12	0.41	0.73
E1-3	508.36	86.41	0.13	489.45	33.44	31.04	125.65	0.23	0.52
E2-1	86.11	24.41	0.02	3.09	7.83	11.73	11.51	0.47	1.40
E2-2	7.14	0.84	0.01	0.52	0.64	1.86	0.96	0.10	0.52
E2-3	1018.36	244.05	3.33	50.61	20.78	36.46	84.02	0.37	1.08
E3-1	740.46	197.95	19.97	40.94	24.24	29.89	60.54	0.27	1.99
E3-2	543.99	124.26	15.93	29.12	15.42	24.27	50.34	0.39	0.95
E3-3	1643.23	359.69	20.37	77.42	48.05	32.53	152.46	0.42	2.59

C, control cage 1 to 3; E, 20E cage 1 to 1. Per cage, three pools á three bees were analysed.

Table S3: Comparison of C_t values determined via qPCR between caged bees receiving pollen in their diet analysed in this study (control and 20E group) and hive nurses, hive foragers as well as caged bees not receiving pollen in their diet analysed in (Buttstedt *et al*., 2013). Ct values of control bees in this study are always most similar to hive nurse bees in (Buttstedt *et al*., 2013).

		mrjp1	*mrjp2*	*mrjp3*	*mrjp4*	*mrjp5*	*mrjp6*	*mrjp7*	*mrjp8*	*mrjp9*
C_t values (mean ± SD)	Control	12.2 ± 0.4	14.6 ± 1.3	16.5 ± 1.8	16.2 ± 1.6	16.5 ± 1.3	18.2 ± 0.7	15.8 ± 0.9	24.8 ± 0.4	22.6 ± 0.6
	20E	15.3 ± 2.5	18.0 ± 2.9	24.3 ± 4.7	18.6 ± 2.7	19.6 ± 1.9	20.3 ± 1.8	17.8 ± 2.3	26.3 ± 1.1	24.3 ± 1.4
	$Workers_c$	25.3 ± 0.2	28.6 ± 1.1	27.8 ± 1.5	24.5 ± 1.0	21.8 ± 0.9	20.9 ± 0.5	24.7 ± 0.4	23.3 ± 0.1	20.8 ± 0.7
	Nurses	12.4 ± 0.7	15.0 ± 1.0	15.5 ± 0.6	15.6 ± 0.7	16.7 ± 0.8	18.3 ± 0.2	15.8 ± 0.6	23.8 ± 0.6	22.5 ± 0.7
	Foragers	14.7 ± 1.3	18.4 ± 1.3	23.3 ± 1.5	20.7 ± 2.0	15.8 ± 1.2	15.0 ± 1.0	21.6 ± 1.3	22.8 ± 0.4	20.1 ± 0.2

C_t values for caged workers ($workers_c$) and nurses underlie the relative gene expression published in Buttstedt *et al*. (2013). A direct comparison of gene expression levels between Buttstedt *et al*. (2013) and this study was unfortunately not possible as different reference genes were used. However, in both studies 15 ng cDNA and the very same primers were used for qPCR and C_t values of the reference genes showed a standard deviation below 1 indicating an approximate comparability of determined C_t values.
